# 

**Published:** 1899-12

**Authors:** 


DECEMBER, 1899.

*Vol. XVII.* *No.* **66.**

LIBRARY
Indexed
S. G. O.

### THE BRISTOL MEDICO-CHIRURGICAL JOURNAL

A Quarterly Journal of the Medical Sciences for the West of England and South Wales

*PUBLISHED UNDER THE AUSPICES OF*
*THE BRISTOL MEDICO-CHIRURGICAL SOCIETY.*

LIBRARY
SURGEON GENERAL'S OFFICE
JAN 1-1900

"Scire est nescire, nisi id me

Scire alius sciret."

*Editor:* R. SHINGLETON SMITH, M.D., B.Sc.

WITH WHOM ARE ASSOCIATED

J. MICHELL CLARKE, M.A., M.D., W. H. HARSANT,
B. M. H. ROGERS, B.A., M.D., B.CH., AND JAMES SWAIN, M.S., M.D.

*Assistant-Editor:* L. M. GRIFFITHS.

*Editorial Secretary:* JAMES TAYLOR.

BRISTOL: J. W. ARROWSMITH.
LONDON: J. & A. CHURCHILL, 7 GREAT MARLBOROUGH STREET.

*Price One Shilling and Sixpence.*

